# Blood–brain barrier permeability is associated with different neuroinflammatory profiles in Alzheimer's disease

**DOI:** 10.1111/ene.16095

**Published:** 2023-10-12

**Authors:** Matilde Bruno, Chiara Giuseppina Bonomi, Francesco Ricci, Martina Gaia Di Donna, Nicola Biagio Mercuri, Giacomo Koch, Alessandro Martorana, Caterina Motta

**Affiliations:** ^1^ UOSD Centro Demenze, Policlinico Tor Vergata University of Rome “Tor Vergata” Rome Italy; ^2^ Neurology Unit University of Rome "Tor Vergata" Rome Italy; ^3^ Human Physiology Unit, Department of Neuroscience and Rehabilitation University of Ferrara Ferrara Italy; ^4^ Experimental Neuropsychophysiology Laboratory IRCCS Santa Lucia Foundation Rome Italy

**Keywords:** Alzheimer's disease, blood–brain barrier, cerebrospinal fluid, cytokines, neuroinflammation

## Abstract

**Introduction:**

Inflammation is an important player in Alzheimer's disease (AD), whose effects can be influenced by the blood–brain barrier (BBB). Here, we investigated the relationship between BBB permeability, indicated by cerebrospinal fluid (CSF)/plasma albumin quotient (Qalb), and CSF indexes of neuroinflammation in a cohort of biologically defined AD patients.

**Methods:**

Fifty‐nine consecutive patients with mild cognitive impairment (MCI) or early AD (Mini‐Mental State Examination [MMSE] >22) underwent CSF analysis for inflammatory cytokines (interleukin [IL]‐1β, IL‐2, IL‐4, IL‐5, IL‐6, IL‐7, IL‐8, Il‐10, IL‐12, IL‐13, IL‐17, tumor necrosis factor‐α [TNF‐α], interferon‐γ [IFN‐γ], granulocyte‐monocyte colony‐stimulating factor [GM‐CSF], granulocyte colony‐stimulating factor [G‐CSF]). Using backward stepwise linear regression analysis, we explored the potential influence of each cytokine CSF level on Qalb considering age, sex, and apolipoprotein E (APOE) as covariates.

**Results:**

Higher levels of IL‐4 (*β* = 0.356, 0.005) and IL‐8 (*β* = 0.249, 0.05) were associated with higher Qalb values, while macrophage inflammatory protein‐1α (MIP‐1β) (*β* = −0.274; *p* = 0.032) and TNF‐α (*β* = −0.248; *p* = 0.031) showed a significant negative association with BBB permeability. Age was also positively associated with Qalb (*β* = 0.283; *p* = 0.016).

**Conclusions:**

Despite the overall integrity of the BBB, its permeability could either influence or be influenced by central neuroinflammation, reflected by CSF cytokine levels. This is in line with previous studies that showed that patients with a more intact barrier are those with more prominent neurodegeneration. Our findings suggest that different neuroinflammatory profiles can be associated with different levels of BBB permeability in AD.

## INTRODUCTION

As the world population ages, dementia is increasingly becoming a global health burden. Among the different causes of cognitive impairment, Alzheimer's disease (AD) is the most prevalent, and great efforts in medical research are directed at finding biomarkers that could lead to an early diagnosis or hint at novel therapeutic targets.

From a pathophysiological point of view, AD is characterized by amyloid‐β (Aβ) and phosphorylated tau (p‐tau) accumulation in plaques and neurofibrillary tangles, respectively. These pathological changes result from the interplay of many different mechanisms and, among others, inflammation due to activation of the innate immune system has emerged as an important player, which has been linked to both beneficial and detrimental effects [1].

What are the factors skewing the effects of inflammation is still a matter of debate. Since the brain can no longer be considered an immune‐privileged organ, much attention has been drawn to the blood–brain barrier (BBB) as a possible gateway regulating the passage of immune cells from the peripheral circulation into the central nervous system (CNS) [2]. Even though the BBB undergoes physiological changes with aging, a leakier membrane seems to be a feature of AD, and the result of several factors challenging its integrity [3]. Moreover, higher BBB permeability seems to correlate with more severe cognitive decline and a worse response to therapeutic attempts, despite no consensus having been reached on the mechanisms behind this finding [4].

With the present work, our aim was to investigate the relationship between BBB permeability, reflected by CSF/plasma albumin quotient (Qalb), and CSF inflammation, as measured by levels of inflammatory cytokines, in a cohort of biologically defined AD patients.

## METHODS

### Subject enrolment

We enrolled 65 consecutive patients who had been referred to the UOSD Centro Demenze of “Policlinico Tor Vergata” in Rome due to memory decline, between April 2022 and December 2022. After clinical evaluation, they fulfilled criteria for either amnestic mild cognitive impairment (MCI) [5] or early dementia due to AD [6] with a Mini‐Mental State Examination (MMSE) score >22. On CSF analysis, all patients belonged to the biologically defined AD continuum (i.e., CSF Aβ1‐42 <600 pg/mL).

We excluded patients with recent ischemic stroke (within the last 6 months or radiological evidence of ischemic lesions), Hachinski score >4, CSF Aβ1‐42 >600 pg/mL, chronic inflammatory/autoimmune conditions, signs and symptoms of ongoing infection, or finding elevated C reactive protein levels in blood. Therefore, our sample eventually included 59 patients.

### CSF collection and analysis

All lumbar punctures and biochemical analyses were performed according to standard practice as described elsewhere [7]. Blood samples were also drawn for complimentary analyses. CSF Aβ1‐42, p‐tau, and t‐tau concentrations were determined using a sandwich enzyme‐linked immunosorbent assay (EUROIMMUN ELISA^©^). APOE genotyping was performed by allelic discrimination technology (TaqMan; Applied Biosystems). We used Bio‐Plex Multiplex Cytokine Assay (Bio‐Rad Laboratories), according to the manufacturer's instructions, to dose the following cytokines: interleukin (IL)‐1β, IL‐2, IL‐4, IL‐5, IL‐6, IL‐7, IL‐8, IL‐10, IL‐12, IL‐13, IL‐17, tumor necrosis factor‐α (TNF‐α), interferon‐γ (IFN‐γ), granulocyte‐monocyte colony‐stimulating factor (GM‐CSF), granulocyte colony‐stimulating factor (G‐CSF), macrophage inflammatory protein‐1α (MIP‐1β), and monocyte chemoattractant protein‐1 (MCP‐1). Concentrations of analytes were calculated according to a standard curve and expressed in picograms/milliliter (pg/mL). When concentrations of the analytes were below the detection threshold they were assumed to be 0 pg/mL.

### Statistical analysis

All continuous variables including levels of CSF biomarkers and cytokines, age, and the calculated Qalb were expressed as means±standard deviations.

We used a backward multivariate regression analysis to explore associations between CSF cytokines levels and BBB permeability, considering age, sex, and apolipoprotein E (APOE) genotype (i.e., the presence of at least one ε4 allele) as covariates. Only cytokines with maximum values below the limit of detection of 5% were considered acceptable to be included in the analysis, thus we excluded IL‐2, IL‐5, IL‐12, IL‐13, IL‐17, and IFN‐γ. Finally, we used Pearson's r analysis to assess correlations between cytokines associated with Qalb and AD core biomarkers.

All statistical analyses were performed with JASP^©^ (Version 0.14 Computer Software; JASP Team 2020). Values of *p* < 0.05 were considered statistically significant.

## RESULTS

The study included 59 patients belonging to the AD continuum (A+T+, *n* = 29; A+T−, *n* = 30). Demographic variables and dosages of CSF cytokines levels are reported in Table 1.

**TABLE 1:** Demographic variables and results of dosages of cerebrospinal fluid cytokine levels.

Parameter	Study cohort (*n* = 59)
Sex (F)	64.4%
Age	71.85 ± 6.54
Qalb	6.56 ± 2.86
CSF t‐tau	472.11 ± 304.19
CSF p‐tau	65.74 ± 32.38
CSF Aβ1‐42	373.05 ± 111.23
APOE (ε4)	62.7%
CSF IL‐1β	0.07 ± 0.12
CSF IL‐2	0.18 ± 0.35
CSF IL‐4	0.23 ± 0.20
CSF IL‐5	0.00 ± 0.04
CSF IL‐6	5.99 ± 6.58
CSF IL‐7	8.59 ± 6.97
CSF IL‐8	23.52 ± 9.32
CSF IL‐10	2.88 ± 0.62
CSF IL‐12	0.87 ± 0.96
CSF IL‐13	1.19 ± 1.38
CSF IL‐17	1.13 ± 1.39
G‐CSF	5.85 ± 3.52
GM‐CSF	52.45 ± 26.57
CSF IFN‐γ	0.85 ± 1.29
CSF MCP‐1	220.00 ± 159.08
CSF MIP‐1β	103.62 ± 149.60
CSF TNF‐α	3.22 ± 4.28

Abbreviations: Aβ1‐42, amyloid β 1‐42; APOE, apolipoprotein E; CSF, cerebrospinal fluid; F, female; G‐CSF, granulocyte colony‐stimulating factor; GM‐CSF, granulocyte‐monocyte colony‐stimulating factor; IFN‐γ, interferon‐γ; IL, interleukin; MCP‐1, monocyte chemoattractant protein‐1; MIP‐1β, macrophage inflammatory protein‐1α; p‐tau, phosphorylated tau; Qalb, plasma albumin quotient; TNF‐α, tumor necrosis factor‐α; t‐tau, total tau.

The backward stepwise linear regression explored the potential influence of CSF levels of each cytokine on QAlb. As explained in the Methods section, we included in the model IL‐1β, IL‐4, IL‐6, IL‐7, IL‐8, IL‐10, G‐CSF, GM‐CSF, TNF‐α, MIP‐1β, MCP‐1, as well as age, sex, and APOE genotype as covariates, upon evaluation of collinearity. At each step, variables were excluded based on the highest *p*‐value, representing the least contribution to the model. Eventually, we were able to reduce the candidate variables to five: age, IL‐4, IL‐8, MIP‐1β, and TNF‐α (*R* ^2^ = 0.352, *F* [5, 53] = 5.770, *p* < 0.001) (see Table 2). Higher values of IL‐4 (*β* = 0.356, *p* = 0.005) and IL‐8 (*β* = 0.249, *p* = 0.050) were associated with higher values of Qalb, while CSF levels of MIP‐1β (*β* = −0.274; *p* = 0.032) and TNF‐α (*β* = −0.248; *p* = 0.031) showed a negative association with BBB permeability. Furthermore, age was also positively associated with QAlb (*β* = 0.283, *p* = 0.016).

**TABLE 2:** Results from stepwise multivariate regression with backward entry model.

Model	Variables	*β*	*T*	*p*‐Value	CI lower	CI upper	*R* ^2^ change	*F* change
Model 1	Sex	−0.093	−0.732	0.468	−1737	0.812	0.426	2334
	Age	0.223	1678	0.100	−0.016	0.176		
	IL‐1β	−0.027	−0.148	0.883	−7871	6796		
	IL‐4	0.434	2559	**0.014** *	1129	9495		
	IL‐6	0.172	1167	0.250	−0.045	0.167		
	IL‐7	0.043	0.244	0.808	−0.106	0.135		
	IL‐8	0.233	1510	0.138	−0.020	0.137		
	IL‐10	−0.100	−0.717	0.477	−1463	0.695		
	G‐CSF	−0.199	−1008	0.319	−0.402	0.134		
	GM‐CSF	−0.130	−0.859	0.395	−0.039	0.016		
	MCP‐1	−0.193	−1483	0.145	−0.013	0.002		
	MIP‐1β	−0.163	−1074	0.289	−0.222	0.068		
	TNF‐α	−0.202	−1201	0.236	−1010	0.256		
	APOE	−0.084	−0.643	0.524	−1694	0.875		
Model 10	Age	0.283	2500	**0.016** *	0.020	0.183	−0.017	1390
	IL‐4	0.356	2961	**0.005** **	1407	7315		
	IL‐8	0.249	2007	**0.050** *	0.000	0.126		
	MIP‐1β	−0.274	−2201	**0.032** *	−0.249	−0.012		
	TNF‐α	−0.248	−2217	**0.031** *	−0.884	−0.044		

*Note*: Bold values represent significativity.

Abbreviations: APOE, apolipoprotein E; CI, confidence interval; CSF, cerebrospinal fluid; G‐CSF, granulocyte colony‐stimulating factor; GM‐CSF, granulocyte‐monocyte colony‐stimulating factor; IFN‐γ, interferon‐γ; IL, interleukin; MCP‐1, monocyte chemoattractant protein‐1; MIP‐1β, macrophage inflammatory protein‐1α; TNF‐α, tumor necrosis factor‐α.

* *p* < 0.05.

** *p* < 0.01.

No association was found between TNF, IL‐4, IL‐8, and MIP‐1β and AD core biomarkers (*p* > 0.05), except for a trend of positive correlation between MIP‐1α and both t‐tau (*p* = 0.058) and p‐tau (0.088).

## DISCUSSION

Inflammation is known to be involved in the pathogenesis of AD, although its role remains unclear. It has been demonstrated that neuroinflammation can exert both detrimental and positive effects, on the one hand leading to the exacerbation of Aβ accumulation, but on the other reducing AD pathological burden [8]. This dualism is further supported by different modalities of phenotypical activation of astrocytes and microglia that could produce either proinflammatory or protective cytokines, depending on disease stage or other complementary factors such as APOE [7] and BBB integrity [2].

Our study addressed the issue of the interplay between BBB permeability and inflammatory cytokine levels in the CSF. First, we found that, within our sample, Qalb mean levels were in range, meaning that the BBB was not disrupted. However, we identified that different degrees of permeability seem to impact neuroinflammation. Indeed, our analysis showed that a leakier BBB is associated with higher levels of IL‐4 and IL‐8, while a tighter BBB was associated with higher TNF‐α and MIP‐1β levels. Moreover, while there was no statistically significant association between these cytokines and AD core biomarkers, we found a positive correlation trend between MIP‐1α and both p‐tau and t‐tau CSF levels.

These results are open to different interpretations. Interestingly, both IL‐4 and IL‐8 have neuroprotective effects: IL‐4 overexpression results in the attenuation of Aβ pathology in animal models [9], and IL‐8 is a microglia‐derived chemokine implicated in hypoxemic‐induced neoangiogenesis [10], which could also cause alterations in BBB permeability. On the contrary, TNF‐α is a proinflammatory cytokine whose increased levels have been linked to the conversion from MCI to AD dementia [11] and MIP‐1β promotes inflammation via chemotaxis [12].

A first hypothesis could see the BBB as an actual gatekeeper, regulating the passage of cytokines in and out of the CSF through changes in its permeability. However, in stages of the disease in which inflammation is skewed towards damage‐repairing effects – reflected by higher levels of neuroprotective cytokines – a more permeable BBB could assist in the clearance of misfolded proteins. This is in line with previous studies correlating BBB breakdown to lower p‐tau in the CSF [13], and with findings that soluble p‐tau fully mediates amyloid‐related tau aggregation in the brain [14]. Thus, a tighter BBB could be associated with more intense neurodegeneration [13], likely sustained by the detrimental contribution of proinflammatory cytokines. The positive trend of correlation between MIP‐1β and t‐tau levels partially supports this hypothesis. However, the small sample size limited our statistical approach and did not allow us to define the causality of association between our variables. Despite not being conclusive, our results add interesting insights on the interplay that ties inflammatory mediators to BBB permeability in AD, which has important prognostic implications since both have been linked to the worsening of cognitive performance [15]. Longitudinal data are needed to clarify whether these two processes have converging or synergistic effects, in order to identify possible intervention strategies aimed at slowing disease progression.

## AUTHOR CONTRIBUTIONS

**Matilde Bruno:** Data curation; visualization; writing – original draft. **Chiara Giuseppina Bonomi:** Writing – original draft; visualization; validation. **Francesco Ricci:** Formal analysis. **Martina Gaia Di Donna:** Validation. **Nicola Biagio Mercuri:** Supervision. **Giacomo Koch:** Funding acquisition; resources; supervision. **Alessandro Martorana:** Supervision; conceptualization; funding acquisition. **Caterina Motta:** Conceptualization; investigation; writing – review and editing; data curation; methodology.

## CONFLICT OF INTEREST STATEMENT

The authors declare that they have no competing interests.

## Data Availability

The data that support the findings of this study are available from the corresponding author upon reasonable request.
